# Preferential crosstalk between perifollicular capillary vessels and dermal papilla cells during hair cycling homeostasis

**DOI:** 10.1038/s41598-026-46001-2

**Published:** 2026-04-01

**Authors:** Ying Zeng, Akinari Abe, Satsuki Takashima, Miyu Kono, Reina Kagiyama, Mariko Komabayashi-Suzuki, Mariko Moriyama, Hiroyuki Moriyama, Tadashi Okamoto, Hideya Ando, Masaki Tanaka, Masamitsu Ichihashi, Masatsugu Ema, Ken-ichi Mizutani

**Affiliations:** 1 Laboratory of Stem Cell Biology, Graduate School of Pharmaceutical Sciences, Kobe Gakuin University, 1-1-3 Minatojima, Chuou-Ku, Kobe, 650-8586 Japan; 2 Self-Medication R&D Laboratories, Taisho Pharmaceutical Co., Ltd., Saitama, 331-9530 Japan; 3 Department of Stem Cells and Human Disease Models, Research Center for Animal Life Science, Shiga University of Medical Science, Otsu, Shiga 520-2192 Japan; 4 Pharmaceutical Research and Technology Institute, Kindai University, Osaka, 577-8502 Japan; 5 Faculty of Pharmaceutical Sciences, Kobe Gakuin University, Kobe, 650-8586 Japan; 6 Faculty of Life Science, Department of Bioscience, Okayama University of Science, Okayama, 700-0005 Japan; 7 Syuhakukai Medical Corporation, Tokyo, 105-0004 Japan; 8 Institute for the Advanced Study of Human Biology (ASHBi), Kyoto University, Kyoto, 606-8501 Japan

**Keywords:** Perifollicular capillary vessels, Vascular remodeling, Dermal papilla, Vascular niche, Hair follicle, Cell biology, Diseases, Physiology

## Abstract

The tissue-specific capillary system supports the unique function of each organ and dynamically remodels in response to local requirements. In this study, we found that perifollicular vascularization flexibly adjusts to accommodate physiological changes. Notably, not all capillary vessels responded and migrated equally. However, those around the dermal papilla (DP) exhibited preferential mobilization. Treatment with minoxidil, a hair growth agent, significantly increased perifollicular vessel mobilization around the DP, whereas it was inhibited in experimental models of tissue aging, such as those involving vascular endothelial growth factor-neutralizing antibody or testosterone treatment, similar to the physiological tissue aging process. Furthermore, vascular endothelial cells triggered the expression of angiogenic chemokine molecules, including CC chemokine ligand 2 (CCL2), in DP cells, and signaling improved crosstalk between perifollicular vessels and the DP. CCL2 expression changed cyclically in the DP vicinity and significantly decreased in aged skin, and treatment with CCL2-neutralizing antibody decreased perifollicular vascularization and suppressed DP function. These findings indicate that the crosstalk between perifollicular vessels and the DP contributes to hair cycling homeostasis and aging, providing a potential target for the treatment of hair loss and other degenerative skin disorders.

**Supplementary Information:**

The online version contains supplementary material available at 10.1038/s41598-026-46001-2.

## Introduction

Adult mammalian skin and hair follicles contain multiple stem and progenitor cell populations, which are crucial for tissue regeneration. The coordinated interaction between epithelial hair follicle stem cells residing in the bulge (Bg)^1^ and mesenchymal cells located at the base of the hair follicle, known as the dermal papilla (DP), plays an important role in the morphogenesis and regeneration of hair follicles. The hair growth cycle includes three phases: anagen (rapid growth), catagen (regression), and telogen (resting period). The number of DP cells (DPCs) fluctuates throughout the cycle. However, the mechanisms underlying the maintenance of the number of DPCs in healthy follicles remain unclear. DPCs stimulate anagen phase initiation by providing instructive signals required to induce epithelial Bg cell proliferation^2^. When the number of DPCs decreases, the initiation of the anagen phase is delayed, and the telogen phase is maintained^3^. Hair thinning and loss have been reported to be partly related to the number of DPCs^4^, which are essential for initiating the anagen phase and preventing hair loss.

Angiocrine and angiogenic factors mediate the communication between vascular endothelial cells (ECs) and their surrounding cells during tissue development and repair. The hair follicle is surrounded by capillary vessels arising from a small set of capillaries near the DP^5^. These vessels nourish the hair follicle by delivering nutrients and oxygen, removing waste products from tissues, and establishing spatiotemporal vascular niches during development, homeostasis, and regeneration. However, these functions are impaired with aging^6–9^. Recently, perifollicular angiogenesis has been reported to be associated with hair cycle homeostasis^10–14^. Vascular endothelial growth factor (VEGF) plays an important role in regulating hair follicle vascularization and is expressed in DPCs, outer root sheath keratinocytes, and ECs. VEGF expression is upregulated during the anagen phase, and transgenic mice with increased cutaneous VEGF levels have been reported to have larger hair follicles than their wild-type littermates^11^. The vascular annulus surrounds the upper Bg throughout the hair cycle and forms a specialized perivascular niche for hair follicle stem cells^13^. Skin vasculature dysplasia is associated with several human diseases characterized by hair loss^15,16^. Alopecia can arise from a shortened hair cycle, resulting in thinner and shorter new hairs. Additionally, it is characterized by decreased perifollicular vascularization and downregulation of vascularization-related genes in balding scalps^17^. A recent study has shown a reciprocal interaction between ECs and DPCs, where ECs release signaling molecules to activate DPCs, which induce hair regeneration, whereas DPCs emit a signal to activate ECs and promote angiogenesis^18^. However, these interactions decrease in aged skin. The primary mechanism of action of minoxidil (MD), a hair growth-promoting drug, is likely mediated by the induction of VEGF production, which stimulates hair follicle vascularization^19^. These findings indicate that effective crosstalk between hair follicles and perifollicular vessels contributes to the morphogenesis and growth of hair follicles. However, the physiological role of spatiotemporal perifollicular vascular remodeling remains unknown.

Therefore, this study aimed to examine how perifollicular vascularization flexibly changes in a spatiotemporally restricted manner to adapt to physiological changes. Particularly, this study investigated the vascular remodeling around DPCs during the hair cycle, aging, and treatment with a VEGF-neutralizing antibody or MD and testosterone (TST) and the physiological significance of crosstalk between DPCs and ECs in proper skin homeostasis.

## Results

### Changes in the distribution of capillaries around the DP based on hair cycle and aging

In this study we decided to utilize *VEGFR1*-tandem dsRed (*Flt1*-tdsRed) mice line to label only vascular ECs, however VEGFR1^+^ cells are a subset of vascular ECs, in combination with CD31 immunostaining, a marker of total ECs. This approach provides a more precise understanding of the blood vessel dynamics surrounding hair follicles.

**Fig. 1:**
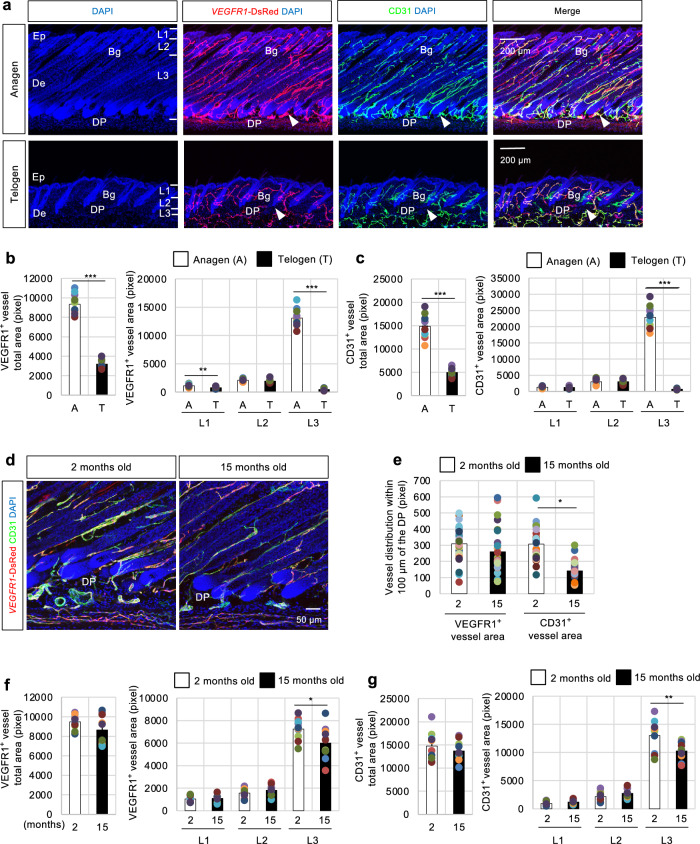
The capillaries surrounding the dermal papilla (DP) undergo preferential changes during the hair cycle and aging. (**a**) Immunofluorescence of CD31 (green) in *VEGFR1*-tdsRed (red) dorsal skin. The vascular pattern of at least 20 murine tissues analyzed during the anagen and telogen phases was typical. Higher magnification is shown in Supplemental Fig. 1a. Scale bar = 200 μm. (**b**, **c**) Quantification of vascular distribution as detected by VEGFR1- (**b**) and CD31- (**c**) positive cells within the epidermis and dermis tissues divided into three regions from the epidermal surface to the dermal papilla (DP) base at both anagen (A) and telogen (T) phases (Layer 1 [L1]: vicinity of infundibulum and isthmus; Layer 2 [L2]: vicinity of bulge (Bg); Layer 3 [L3]: vicinity of DP); 15 independent sections from 3 individual murine dorsal skin samples. Data were presented as mean ± SEM. T-test, **p < 0.01, ***p < 0.001. (**d**) Analysis of the vascular pattern in *VEGFR1*-tdsRed dorsal skin at 2 and 15 months of age. The arrowhead indicates the preferential decreased vascularization in aged tissue around the DP; 13 independent sections from 3 individual murine dorsal skin samples. Scale bar = 50 μm. (**e**) Comparison of VEGFR1- or CD31-positive cells distributed within 100 µm of the DP between 2 and 15 months of age. (**f**, **g**) Quantification of vascular distribution as detected by VEGFR1- (**f**) and CD31- (**g**) positive cells within the epidermis and dermis tissues divided into three regions from the epidermal surface to the DP base (Layer 1 [L1]: vicinity of infundibulum and isthmus; Layer 2 [L2]: vicinity of Bg; Layer 3 [L3]: vicinity of DP); 13 independent sections from 3 individual murine dorsal skin samples. Data were presented as mean ± SEM. T-test, *p < 0.05.

First, blood vessel localization during the hair cycle and aging was carefully observed to clarify the role of perifollicular capillary vessels around the DP. Dorsal skin sections of *VEGFR1*-tdsRed mice were analyzed during the anagen (postnatal day 33 [P33]) and telogen (P44) phases of the physiological postnatal natural hair cycle to investigate the localization of capillary vessels, with vessels marked by DsRed and the panvascular marker CD31 (also known as PECAM1). During the natural hair cycle, the skin vasculature was remarkably remodeled during the anagen phase, and capillary vessels were abundant and located close to the entire hair follicle (Fig. 1a arrowhead and Supplementary Fig. 1a). Conversely, the number of perifollicular capillary vessels surrounding the DP decreased during the telogen phase. However, other capillaries remained in the upper Bg (Fig. 1a and Supplementary Fig. 1a). Similar findings were observed during depilation-induced synchronized adult hair cycling (Supplementary Fig. 1c). Furthermore, the distribution of capillaries was quantified (Supplementary Fig. 1 d) to further characterize capillary vessel localization in relation to hair follicles (Fig. 1b,c). The distributed capillaries were preferentially concentrated around the DP during the anagen phase (L3). In this analysis to evaluate capillary distribution within the tissue, vascular area (pixel) was used. However, when comparing the anagen phase and telogen phase, the tissue area undergoes drastic changes, potentially leading to overestimation of the quantitative values. Therefore, vascular percentage (%) was also quantified (see Materials and methods for details). Results similarly confirmed increased distribution in L3 during the anagen phase (Supplementary Fig. 1b).

Second, the vascular networks of young (2 months old) and older tissues (15 months old) were compared using the dorsal skin of *VEGFR1*-tdsRed mice (Fig. 1d and Supplementary Fig. 2a) to investigate the aging of capillaries around hair follicles. Quantification data showed that the total capillary density slightly decreased in older mice. However, no statistically significant difference was observed (Fig. 1f,g). Nevertheless, the localization of the regular vascular patterning was altered in the hair follicles of older mice. Notably, vascular density was significantly reduced in L3 in the older group (Fig. 1d) compared with that in the younger group, as determined by VEGFR1- and CD31-positive areas (Fig. 1f,g). However, the capillary densities of L1 and L2 did not exert a significant effect. Consistent with this phenomenon, a significant reduction in skin layer thickness was also observed at L3 (Supplementary Fig. 2b). Furthermore, the distribution of capillaries around the DP was investigated, and the vascular density within 100 µm of the DP was analyzed. The distribution of blood vessels near the DP was significantly reduced in the older group (Fig. 1e). Such selective changes in the capillary vessels surrounding the DP indicate that a biologically responsive population within perifollicular capillary vessels contributes to maintaining hair follicle tissue homeostasis.

### VEGF signaling regulates perifollicular niche

To evaluate the effects of the VEGF neutralizing antibody bevacizumab (Bev) and other agents on the perifollicular capillary vessels, we focused on the telogen phase, which allows for more reliable synchronization between animals. Because the perifollicular vasculature undergoes dynamic changes throughout the hair cycle, it is necessary to assess its density at the same time point in the hair cycle across different mice. While accurately synchronizing anagen hair follicles is technically challenging, especially when drug administration like Bev may inhibit hair cycle progression, we set the sampling day by using changes in dorsal skin at the onset of telogen as an indicator (see Materials and methods for details).

**Fig. 2:**
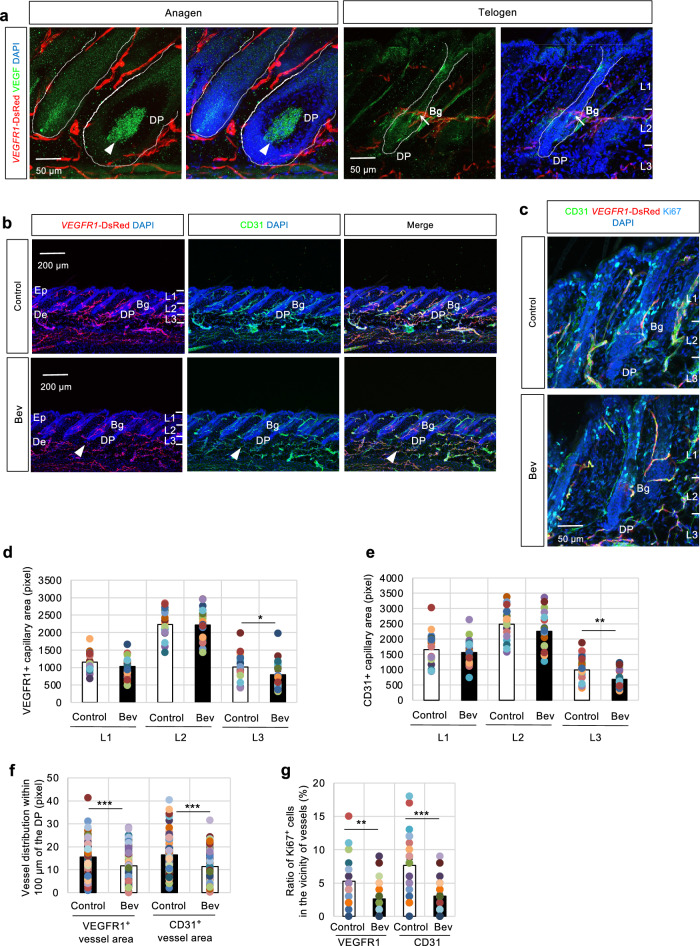
The capillaries surrounding the dermal papilla (DP) undergo preferential regulation by treatment with a VEGF-neutralizing antibody (bevacizumab; Bev). (**a**) Immunofluorescence of VEGF in *VEGFR1*-tdsRed dorsal skin. Distinct vascularization around the dermal papilla (DP) dependent on the hair cycle, consistent with the VEGF (green) expression level. The arrowhead indicates the strong VEGF expression around the DP at the anagen phase, whereas the arrow denotes the weak expression level around the bulge (Bg) during the telogen phase. The vascular pattern of at least 20 murine tissues analyzed during the anagen and telogen phases was typical. Scale bar = 50 μm. (**b**, **c**) Treatment of *VEGFR1*-tdsRed mice with VEGF-neutralizing antibody (Bev, 5 mg/kg, concentration at 0.5 mg/ml) or vehicle (Control) daily at 9 AM for 6 days intraperitoneally during the telogen phase, followed by immunostaining with CD31 (green) (**b**, **c**) and Ki67 (cyan blue) (**c**) during the telogen phase; 20 independent sections from 3 individual murine dorsal skin samples. b, scale bar = 200 μm; c, scale bar = 50 μm. (**d**, **e**) Quantification of vascular distribution as detected by VEGFR1- (d) and CD31- (e) positive cells within the epidermis and dermis tissues divided into three regions from the epidermal surface to the DP base (Layer 1 [L1]: vicinity of infundibulum and isthmus; Layer 2 [L2]: vicinity of Bg; Layer 3 [L3]: vicinity of DP); 10 independent sections from 3 individual murine dorsal skin samples. Data are presented as mean ± SEM. T-test, *p < 0.05, **p < 0.01. (**f**) Analysis of VEGFR1- or CD31-positive cells distributed within 100 µm of the DP in Bev-treated dorsal skin tissues. Data are presented as mean ± SEM. T-test, ***p < 0.001. (**g**) Quantification of the ratio of Ki67-positive cells in the vicinity of capillary vessels, calculated from 8 independent sections from 3 individual murine dorsal skin samples. Data are presented as mean ± SEM. T-test, **p < 0.01, ***p < 0.001.

VEGF signaling is crucial for physiological and pathological angiogenesis^20,21^, and VEGF signaling insufficiency affects aging in various tissues^9^. During the natural hair cycle, abundant VEGF proteins were found in the DP at the anagen phase (Fig. 2a, arrowheads), although the expression was dramatically attenuated and weak VEGF expression was maintained mainly around the Bg at the telogen phase (Fig. 2a, arrows). Therefore, the influence of VEGF impairment on perifollicular vessel homeostasis was investigated. *VEGFR1*-tdsRed mice were treated with the VEGF-neutralizing antibody bevacizumab (Bev) daily for 6 days, and immunostaining was performed (Fig. 2b,c). Bev is a humanized monoclonal antibody against human VEGF-A that is extensively used to inhibit angiogenesis^22^. Bev treatment significantly decreased the capillary density of L3 (Fig. 2b arrowhead, d and e). However, it did not exert a significant effect on the capillary density of L1 and L2 (Fig. 2d,e). Furthermore, the vascular density within 100 µm of the DP was analyzed. The results confirmed that Bev treatment significantly reduced the distribution of blood vessels near the DP (Fig. 2f). Therefore, VEGF signaling downregulation showed a preferential decrease in capillary vessels in the DP vicinity.

The perivascular microenvironment contributes to the maintenance of stem cells. Stem and progenitor cells preferentially localize near vessels in various tissues, including the bone marrow^23^, testes^24^, and brain^25,26^. Immunofluorescence analysis was performed using Ki67 (Fig. 2c) to test whether perivascular reduction can cause the loss of stem and progenitor cell functions in the hair follicle as in several tissues^27–29^. The ratio of Ki67-positive cells in the vicinity of these vessels in Bev-treated tissues decreased dramatically in VEGFR1- and CD31-positive vessels (Fig. 2g). On the other hand, the older dorsal skin exhibited a significantly decreased number of Ki67-positive cells in the hair follicle tissue in both anagen (around the DP) and telogen (around the Bg) phases (Supplementary Fig. 2 d) in parallel with a decrease of periventricular vessels. Given that the vascular environment is strongly associated with hair follicle function, the ability of the vascular niche to regulate paracrine factors that activate DPCs was investigated. Conditioned media (CM) were collected from cultured human umbilical vein endothelial cells (HUVECs), and the effects of CM (EC-CM) on cultured human follicle dermal papilla cells (HFDPCs) were investigated (Supplementary Fig. 3a-d). Incubation with EC-CM significantly increased DPC proliferation and Ki67-positive cell percentage (Supplementary Fig. 3b,c) compared with incubation with equivalent amounts of control-CM. In addition, DPCs with EC-CM increased VEGF and ALP mRNA levels (Supplementary Fig. 3d)^30^. Therefore, vascular ECs may preferentially activate DPCs. These findings indicate that the appropriate localization of perifollicular capillary vessels influences the vascular niche that regulates DP function.

### MD enhances vascular remodeling near the DP

**Fig. 3:**
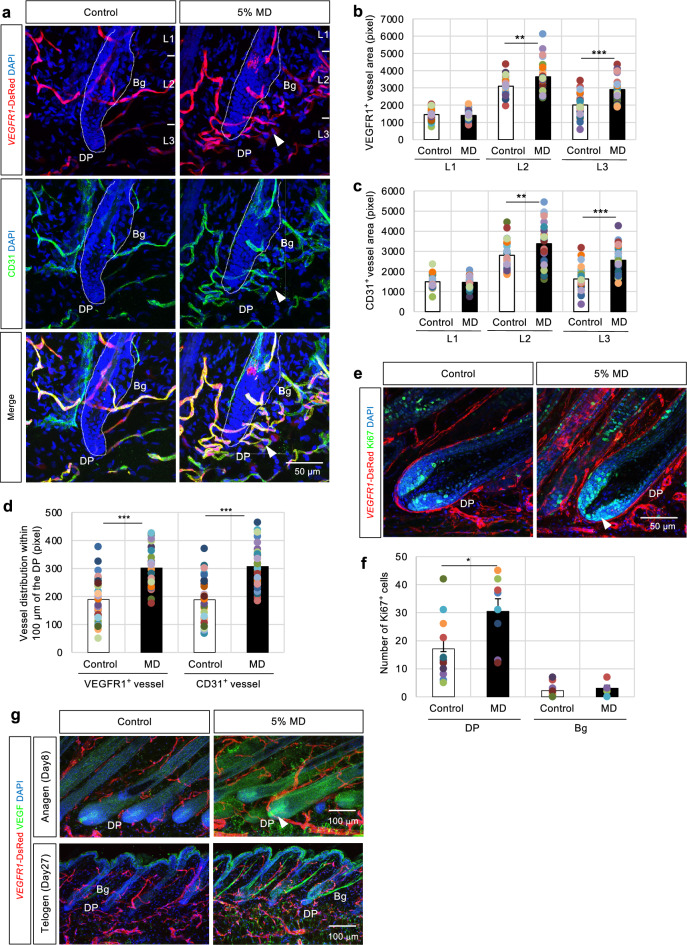
Topical minoxidil (MD) application enhances vascular remodeling near the dermal papilla (DP). (**a**) Treatment of *VEGFR1*-tdsRed mice with 100 μl 5% MD solution or vehicle (Control) daily for 27 days, followed by immunostaining with CD31 (green) during the telogen phase. Higher magnification in the lower panel (merge view); 28 independent sections from 4 individual murine dorsal skin samples. Scale bar = 50 μm. (**b**, **c**) Quantification of vascular distribution as detected by VEGFR1- (**b**) and CD31- (**c**) positive cells within the epidermis and dermis tissues divided into three regions from the epidermal surface to the dermal papilla (DP) base (Layer 1 [L1]: vicinity of infundibulum and isthmus; Layer 2 [L2]: vicinity of bulge (Bg); Layer 3 [L3]: vicinity of DP); 10 independent sections from 3 individual dorsal skin samples. Data are presented as mean ± SEM. T-test, **p < 0.01, ***p < 0.001. (d) Analysis of VEGFR1- or CD31-positive cells distributed within 100 µm of the DP in MD-treated dorsal skin tissues. Data are presented as mean ± SEM. T-test, ***p < 0.001. (**e**) Immunofluorescence of Ki67 (green) in *VEGFR1*-tdsRed (red) dorsal skin after continuous treatment with 100 μl 5% MD solution for 8 days. Scale bar = 50 μm. (**f**) Quantification of Ki67-positive cells in the vicinity of capillary vessels around the DP and Bg, calculated from 8 independent sections from each individual murine dorsal skin sample. (**g**) Immunofluorescence of VEGF (green) in *VEGFR1*-tdsRed (red) dorsal skin after treatment with continuous 5% MD solution for 8 days (upper panel; anagen phase) or for 27 days (lower panel; telogen phase). Scale bar = 100 μm.

The potential of MD to directly enhance vascular mobilization and remodeling around hair follicles was investigated in vivo. MD was continuously topically applied in 7-week-old mice with synchronized depilation. Briefly, after 3 days of depilation, 5% MD was topically applied daily for 27 days, thick sections of the dorsal skin were prepared, and capillary vessel localization was examined by immunofluorescence staining. After continuous MD treatment, the capillary vessels were dramatically mobilized around the hair follicles (Fig. 3a arrowhead). Particularly, the characteristic horizontal capillaries were adjacent to the DP (Fig. 3a). Quantification data showed that the MD treatment significantly increased the capillary density of L2 and L3, but the change in L3 was more prominent. However, no significant effect was observed on L1 (Fig. 3b,c). Additionally, the vascular density within 100 µm of the DP was analyzed. MD treatment significantly increased the number of blood vessels near the DP (Fig. 3d). These findings indicate that MD treatment significantly induces vascular localization around the DP. These findings demonstrate that MD treatment significantly induces the localization of vascular ECs around the DP, while confirming that no changes were observed in lymphatic vessels in this experimental system (Supplementary Fig. 2e).

Next, 5% MD was topically applied daily for 8 days, and the localization of both Ki67-positive cells and capillary vessels around the DP at the anagen phase was investigated. Interestingly, MD treatment significantly increased the number of Ki67-positive cells in the vicinity of vessels near the DP (Fig. 3e arrowhead, f). However, no such changes were observed around the Bg (Fig. 3f). Additionally, MD treatment increased VEGF expression around the Bg, but strongly increased its expression at the hair follicle tip, predominantly in the DP (Fig. 3g arrowhead) where induced angiogenesis was most prominent (average VEGF expression intensity around the region in the 5% MD treatment at anagen phase for DP = 269.2 ± 63.3 and Bg = 70.0 ± 27.9). These findings indicate that MD may contribute to vascular mobilization around the hair follicle tip and confers an appropriate vascular niche, thereby regulating DPCs.

### TST treatment suppresses vascular remodeling near the DP

**Fig. 4:**
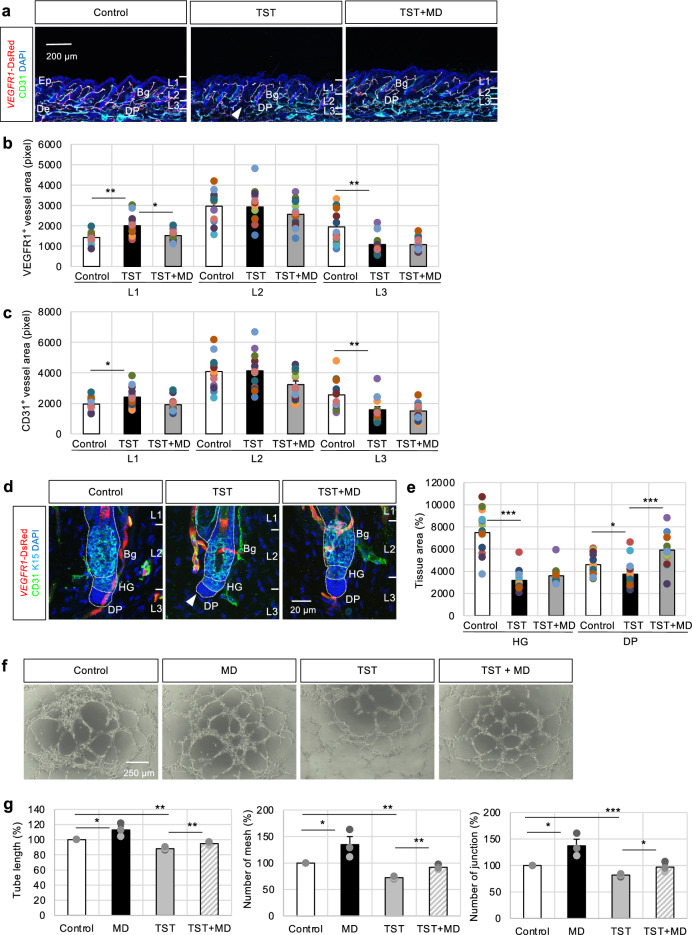
Topical testosterone (TST) application suppresses vascular remodeling near the dermal papilla (DP). (**a**) Treatment of *VEGFR1*-tdsRed mice with 100 μl 0.05% testosterone (TST) or 100 μl 0.05% TST with 100 μl 5% MD daily for 25 days, followed by immunostaining with CD31 (green) during the telogen phase. (Ep, epidermis; De, dermis); 14 independent sections from 6 individual murine dorsal skin samples. Scale bar = 200 μm. Quantification of VEGFR1- (**b**) or CD31-positive (**c**) vascular distribution in dorsal skin tissues within the epidermis and dermis tissues divided into three regions from the epidermal surface to the dermal papilla (DP) base (Layer 1 [L1]: vicinity of infundibulum and isthmus; Layer 2 [L2]: vicinity of bulge (Bg); Layer 3 [L3]: vicinity of DP); 10 independent sections from 3 individual murine dorsal skin samples. Data are presented as mean ± SEM. One-way ANOVA with Least Significant Difference (LSD) post hoc test, *p < 0.05, **p < 0.01. (**d**) Immunofluorescence of K15 (cyan blue) after continuous treatment with 0.05% TST or 0.05% TST with 5% MD daily for 25 days to determine the tissue areas (**e**) of hair germ (HG) and DP. Scale bar = 20 μm. Data are presented as mean SEM. One-way ANOVA with LSD post hoc test, *p < 0.05, ***p < 0.001. (**f**, **g**) Treatment of human umbilical vein endothelial cells (HUVECs) on Matrigel with MD in the presence or absence of TST for 6 h, followed by a tube formation assay. Representative images (**f**) of the untreated and treated groups, quantified tube length, and number of branch points and junctions. Scale bar = 250 μm. Data are presented as mean ± SEM. One-way ANOVA with LSD post hoc test, *p < 0.05, **p < 0.01, ***p < 0.001.

Androgens, such as TST and dihydrotestosterone, exert their effect on human hair follicles either directly or after conversion by 5α-reductase into dihydrotestosterone, inhibiting the proliferation of DPCs, resulting in shorter hair cycle and hair loss^31^. However, the influence of androgenic alopecia on perifollicular vessels remains unknown. Therefore, the influence of TST on vascular mobilization and remodeling in hair follicles was investigated in vivo. TST, with or without MD, was topically applied daily for 25 days in mice. The TST-treated group demonstrated approximately a 10% reduction in vascular density in L3 (Fig. 4a arrowhead) compared with the control group, as determined by VEGFR1- and CD31-positive areas (Fig. 4a-c). In addition, the ratio of CD34-positive cells in the vicinity of vessels in the TST-treated group was significantly decreased in VEGFR1-positive vessels, but MD could prevent them (Supplementary Fig. 2f). Furthermore, the DP tissue area after TST treatment shrank significantly compared with that after the control treatment (Fig. 4d arrowhead). However, MD treatment recovered the TST-induced reduction (Fig. 4d,e). Therefore, TST treatment preferentially influenced the capillary vessels near the DPCs, resulting in abnormal DP growth and maintenance. However, topical MD administration partially improved TST-induced vascular abnormalities of the stem cell niche. Additionally, TST treatment significantly increased the capillary density of L1, possibly direct action of TST on the capillary vessels, although further investigation is needed to be clarified.

The involvement of vascular mobilization after MD treatment helped determine whether MD directly participates in regulating the angiogenic potential of vascular ECs. The angiogenic activities of MD and TST were investigated at the cellular level. The *in vitro* angiogenesis assay results showed that MD treatment significantly increased the formation and branching of tube-like structures on Matrigel (Fig. 4f), with an associated increment in the tube length and master segments. Conversely, TST treatment significantly inhibited angiogenesis. Furthermore, MD treatment reversed TST-induced angiogenesis abnormalities (Fig. 4g).

These findings highlight the importance of further clarifying the interaction between blood vessels and DPCs, as perifollicular capillary vessels near the DP appear to be the most responsive.

### Vascular EC-derived secretion factors promote DPCs function

**Fig. 5:**
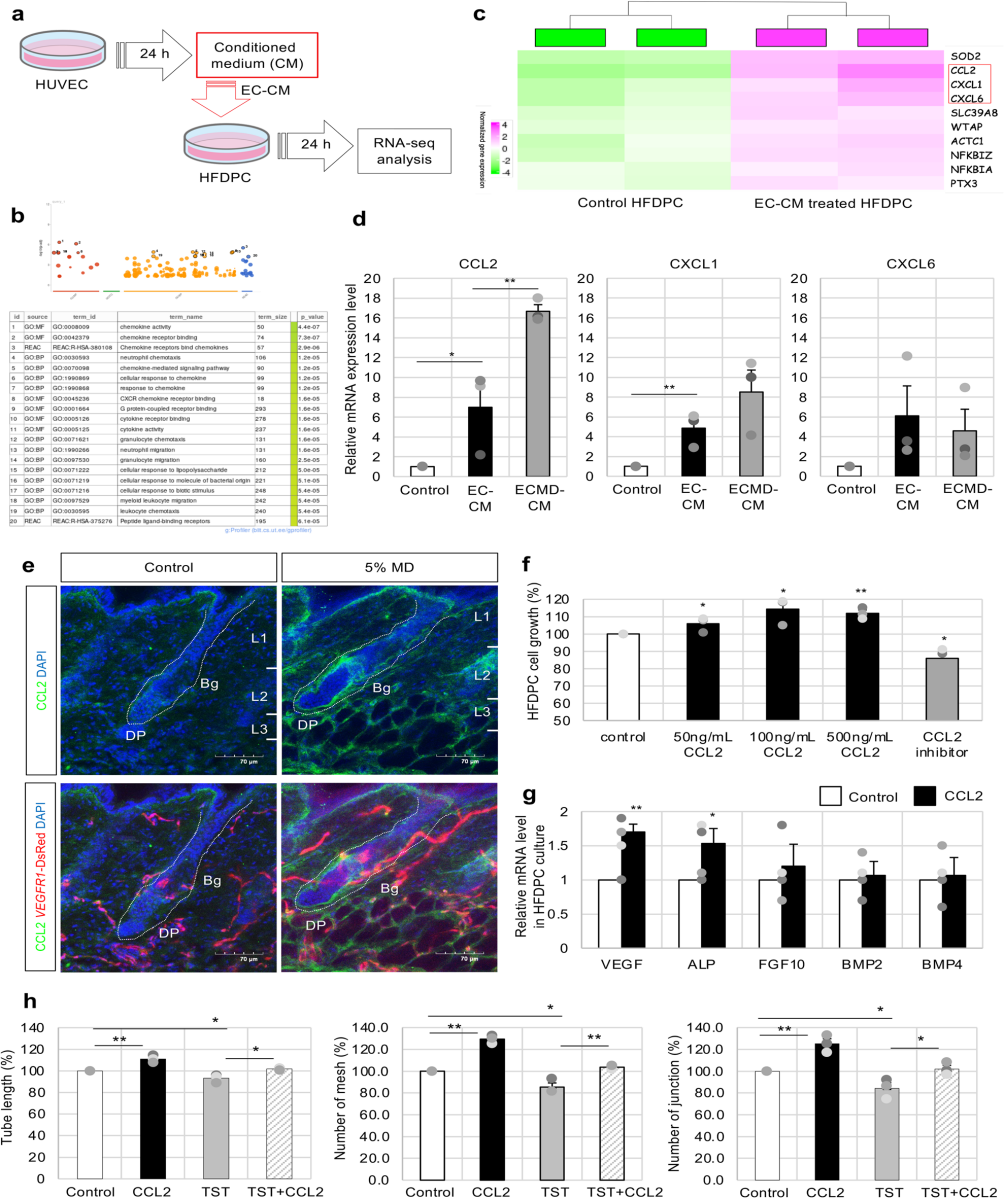
Dermal papilla cell (DPC)-derived chemokine regulates DPCs and the endothelial cells (ECs). (**a**) RNA sequencing analysis of expression profiles of HUVEC-derived conditioned medium (EC-CM)-treated human follicle dermal papilla cells (HFDPCs) and control medium-treated HFDPCs (Control HFDPCs). (**b**) Gene Ontology analysis indicated signaling pathways enriched in HFDPCs treated with HUVEC-CM. (**c**) Changes in some chemokine molecules. (**d**) Comparison of candidate chemokine gene expression among EC-CM-treated HFDPCs, EC in the presence of MD (ECMD)-CM-treated HFDPCs, and control HFDPCs by quantitative polymerase chain reaction (qPCR); n = 3 independent experiments. Data are presented as mean ± SEM. One-way ANOVA with Least Significant Difference (LSD) post hoc test, *p < 0.05, **p < 0.01. (**e**) Immunofluorescence of CCL2 (green) in the dorsal skin of *VEGFR1*-tdsRed (red) mice during the telogen phase after continuous treatment with 100 μl 5% MD solution or vehicle (Control) daily for 27 days. Higher magnification around DPC is shown in Supplemental Fig. 3f. Scale bar = 70 μm. (**f**) Treatment of HUVECs with CCL2 recombinant protein for 24 h, followed by a cell proliferation assay; n = 3 independent experiments. Data are presented as mean ± SEM. One-way ANOVA with LSD post hoc test, *p < 0.05, **p < 0.01. (**g**) Treatment of HUVECs with 100 ng/mL CCL2 recombinant protein for 24 h, followed by qPCR analysis of dermal papilla (DP) function markers; n = 4 independent experiments. Data are presented as mean SEM. T-test *p < 0.05, **p < 0.01, ***p < 0.001. (**h**) Treatment of HUVECs on Matrigel with CCL2 recombinant protein in the presence or absence of TST for 6 h, followed by a tube formation assay. Representative images of the untreated and treated groups, quantified tube length, and number of branch points and junctions were shown in Supplemental Fig. 3a; n = 3 independent experiments. Data are presented as mean ± SEM. One-way ANOVA with LSD post hoc test, *p < 0.05, **p < 0.01.

The transcriptional changes in the HFDPC culture induced by EC-CM were investigated using RNA sequencing to explore the molecular mechanism underlying the crosstalk between ECs and DPCs (Fig. 5a). EC-CM was collected from HUVEC cultured in EGM-2 medium for 24 h, which contains endogenous angiogenic factors. Control media were prepared in parallel using the same EGM-2 medium but without HUVEC. The enrichment analysis revealed that the significantly upregulated genes in the EC-CM-treated group were enriched for Gene Ontology terms, including chemokine activity, chemokine-mediated signaling pathway, and cellular response to chemokine (Fig. 5b). Additionally, the top 10 genes significantly associated with EC-CM-treated HFDPCs were curated (Fig. 5c). Some of these key changes were verified by quantitative polymerase chain reaction. Collectively, changes in several chemokine molecules, including CC chemokine ligand 2 (CCL2), were identified in EC-CM-treated HFDPCs (Fig. 5d). EC-CM treatment combined with MD (ECMD-CM) significantly increased the mRNA expression level of *CCL2* compared with CM treatment alone or control treatment (Fig. 5d), and immunofluorescence staining data for CCL2 in HFDPC culture also supported this result (Supplementary Fig. 3e). Furthermore, the expression of CCL2 was specifically localized in hair follicles including DPCs, and its expression level was significantly promoted by MD treatment (Fig. 5e and Supplementary Fig. 3f).

The CC family of chemokines has been implicated in the cell mobilization ability of stem cells^32,33^. However, their functions in DPCs remain poorly understood. Therefore, HFDPCs were cultured in the presence of CCL2 recombinant protein, and the proliferation and expression levels of DP marker genes were determined. The CCL2-containing medium significantly improved HFDPC proliferation (Fig. 5f) and the expression levels of some marker genes, such as VEGF and ALP (Fig. 5g). Furthermore, TST treatment significantly decreased angiogenesis, as shown in the tube formation assay (Fig. 4g), whereas CCL2 treatment reversed TST-induced angiogenesis abnormalities (Fig. 5h, Supplementary Fig. 3a). These findings indicate that the accumulation of vascular ECs induces CCL2 expression in DPCs and that this signal transduction promotes DP function and contributes to promoting angiogenesis by acting on surrounding ECs.

### CCL2 regulates the crosstalk between the DP and the perifollicular vessels

**Fig. 6:**
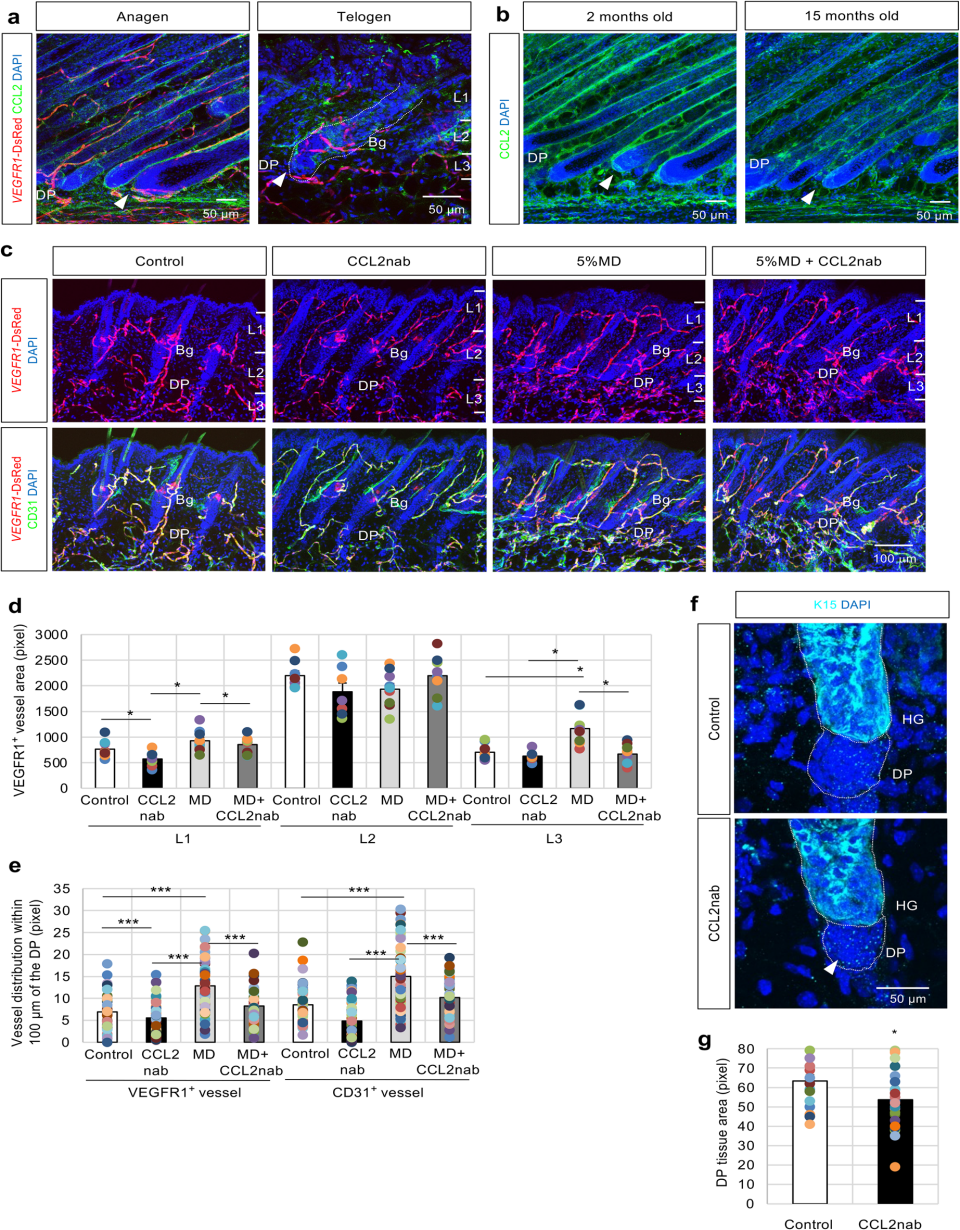
CCL2 impairment influences endothelial cell (EC) and dermal papilla (DP) function. **(a**) Immunofluorescence of CCL2 (green) in *VEGFR1*-tdsRed (red) dorsal skin at the anagen and telogen phases. Scale bar = 50 μm. (**b**) Immunofluorescence of CCL2 (green) in *VEGFR1*-tdsRed dorsal skin at the anagen phase at 2 and 15 months of age. Scale bar = 50 μm. (**c**) Topical application of 100 μl vehicle (Control) or 5% MD on the back skin of *VEGFR1*-tdsRed (red) mice for 25 days, with or without 60 μg intradermal administration of CCL2-neutralizing antibody (CCL2nab) every three days, followed by immunostaining with CD31 (green); 9 independent sections from 3 individual murine dorsal skin samples. (**d**) Quantification of VEGFR1-positive vascular distribution in dorsal skin tissues within the epidermis and dermis tissues divided into three regions from the epidermal surface to the dermal papilla (DP) base (Layer 1 [L1]: vicinity of infundibulum and isthmus; Layer 2 [L2]: vicinity of bulge (Bg); Layer 3 [L3]: vicinity of DP); 10 independent sections from 3 individual dorsal skin samples. Data are presented as mean ± SEM. One-way ANOVA with Least Significant Difference (LSD) post hoc test, *p < 0.05. (**e**) Analysis of VEGFR1- or CD31-positive cells distributed within 100 µm of the DP in CCL2nab-treated dorsal skin tissues. Data are presented as mean ± SEM. One-way ANOVA with LSD post hoc test, ***p < 0.001. (**f**) Immunofluorescence of K15 (cyan blue) after continuous treatment with 60 μg CCL2nab daily every three days for 25 days to determine the HG and DP tissue areas (**g**). Scale bar = 50 μm. Data are presented as mean ± SEM. T-test, *p < 0.05.

CCL2 expression levels in tissues subjected to hair cycle changes, aging changes, and Bev administration were investigated to clarify the importance of CCL2 in hair follicles. CCL2 expression in hair follicles increased during the anagen phase and decreased during the telogen phase (Fig. 6a). Additionally, CCL2 expression in aged hair follicles was preferentially downregulated in the DP vicinity (Fig. 6b). Furthermore, Bev-treated hair follicle tissues demonstrated a significant reduction in CCL2 expression near the DP (Supplementary Fig. 3b).

Finally, the effect of CCL2 impairment on both perifollicular vessels and DP function was investigated. CCL2-neutralizing antibodies (CCL2nab) were intradermally injected into 7-week-old mice daily for 27 days, and immunostaining was performed (Fig. 6c). The continuous administration of CCL2nab reduced the density of perifollicular capillary vessels (Fig. 6c,d and Supplementary Fig. 3 d,e). The capillaries surrounding the DP were most affected (Fig. 6e). Additionally, CCL2nab significantly decreased the DP tissue area (Fig. 6f arrowhead and g), consistent with the findings indicating decreased DPC proliferation by CCL2 inhibitor (Fig. 5f). These findings indicate that blood vessels localized near the hair follicle tip are closely related to functional changes in the DP, and CCL2 contributes to regulating their crosstalk.

## Discussion

Although the tissue microenvironment is important for stem cell function, the molecular signals controlling the dynamics of skin vasculature and mechanisms underlying the changes in these vessel growth patterns during the hair cycle, aging, and MD treatment remain unknown. This study suggested that the spatiotemporal localization of capillary vessels created perifollicular-specific niches that DPCs potentially used for proper skin homeostasis.

This study focused on the spatiotemporal variation of VEGF expression levels in hair follicle tissues. VEGF and its receptors are highly pleiotropic signaling pathway that acts on both vascular^9^ and nonvascular cells such as DP^10,11,34^. In addition to its angiogenic activity, VEGF acts as a survival factor for newly formed blood vessels, maintaining organ-specific vascular traits and inducing certain organ-specific angiocrine factors. In our recent study, the spatiotemporal localization of VEGF during brain development constructed the microenvironment of neural stem cells from the growth state to the differentiation state^25^. VEGF mRNA expression levels in hair follicles have been reported to be temporally upregulated during the anagen growth phase in the induced adult hair cycle and the physiological first postnatal hair cycle^10^. This study confirmed that VEGF expression is spatiotemporally controlled between the anagen phase (abundant VEGF protein predominantly in the DP) and the telogen phase (faint VEGF protein predominantly in the Bg). Additionally, continuous MD treatment did not noticeably influence the whole perifollicular ECs but enhanced angiogenesis near the hair follicle tip, consistent with VEGF expression localization. Therefore, the vascular network surrounding the hair follicle tip may preferentially exhibit remodeling and reconstruction in response to various environmental changes, and capillary mobilization to the DP may regulate DPC functions.

DPCs are specialized mesenchymal cells located in the skin that regulate hair follicle growth and serve as a reservoir of multipotent stem cells^35^. DPCs regulate the hair cycle by secreting growth factors and cytokines. However, the molecular mechanism underlying the crosstalk between DPCs and ECs remains unknown. Recent reports based on single-cell RNA-seq analysis have shown that the reciprocal interaction between DPCs and ECs regulates hair regeneration and angiogenesis^18^. This study showed that perifollicular mobilization near the DP appeared to respond sensitively to physiological changes. Additionally, both aging and treatment with a VEGF-neutralizing antibody or TST decreased capillary vessels near the DP. However, continuous MD application significantly increased them, even during the telogen phase. Furthermore, endothelial vessels appeared to supply signals to strengthen chemokine expression in the DP, thereby stimulating DPC proliferation and function, and MD treatment greatly increased this expression.

Vascular endothelial niches play an extensive role. Tissue-specific ECs mastermind these complex tasks by providing the repopulating cells with stimulatory and inhibitory growth factors, morphogenesis, extracellular matrix, and chemokines. The CC chemokine family has been implicated in stem cell mobilization processes, such as migration, homing, and retention^36,37^. CCL2 has been reported to enhance pluripotency and improve the culture of mouse and human induced pluripotent stem cells^38,39^, and this chemokine factor regulates the self-renewal and proliferation of neural stem cells^40^. Additionally, CCL2 mediates the crosstalk between cancer cells and stromal fibroblasts to control breast cancer stem cells^41^. Furthermore, keratinocyte-derived CCL2 plays an essential role in hair follicle activation^42^, and CCL2 recombinant protein has been reported to increase multipotency and regenerative potential in the skin organoid culture system^43^. Therefore, these findings suggested that CCL2 participates in regulating the multipotency of various tissue stem cells. On the other hand, the CC chemokine family is involved in angiogenesis^44^. CCL2 has been considered an angiogenic and angiocrine chemokine^36,37,45–47^, and VEGF mediates CCL2-induced angiogenesis^37^. This study showed that CCL2 recombinant protein induces VEGF expression in DPCs, and EC-CM stimulated CCL2 expression in DPCs, while VEGF neutralization decreases CCL2 expression, suggesting a positive feedback loop between CCL2 and VEGF in the DP. Incubation with EC-CM significantly increased DPC proliferation and VEGF and ALP expression levels. Therefore, incubation of DPCs with EC-CM treated with MD further increased CCL2 expression in DPCs, and blocking CCL2 with a neutralizing antibody inhibited MD-induced perifollicular vascularization. In addition, our *in vitro* data indicate that CCL2 alone can directly induce angiogenesis independently of VEGF signaling. These findings suggest that CCL2 in the DP is an autocrine regulator of DPC proliferation and function, while also serving as a paracrine mediator that communicates with perifollicular capillary vessels to contributes in angiogenesis through both VEGF-independent and VEGF-dependent ways. Moreover, the inhibition of angiogenesis regarding to TST *in vitro* was reversed by MD and CCL2, suggesting that CCL2 mediates the interaction between MD and TST. While previous study has reported that MD reduces androgen receptor expression^48^, further clarification is needed regarding its relationship with CCL2.

The findings of this study deepen our understanding of vascular remodeling around the hair follicle and provide valuable insights into the treatment of skin disorders, however, several limitations exist in the current study. This study primarily focused on the relationship between indirect interactions mediated by DP and vascular remodeling. Indeed, the induction of CCL2 expression by EC-CM observed in HFDPCs is not observed in keratinocytes or fibroblasts (Supplementary Fig. 3h). Furthermore, it has been confirmed that this interaction between ECs and DP cells is observed in CM derived from vascular ECs, but not CM derived from lymphatic ECs (Supplementary Fig. 3i). Nevertheless, as the direct promotion of angiogenesis by MD treatment clearly demonstrates, understanding vascular changes—including their association with surrounding macrophages or hair follicle epithelial cells—is considered essential for elucidating the dynamic changes within hair follicle tissue. For example, CCR2^45^, the receptor for CCL2, is expressed in macrophages and has been shown to contribute to tissue repair via angiogenesis^49^. Furthermore, our preliminary experiments confirmed that CCR2-positive macrophages undergo hair cycle-dependent changes (data not shown). Although technical constraints made it difficult to evaluate the anagen phase in this study, further investigation into the regulatory mechanisms of DP cells, including the dynamic changes of hair follicle stem cells, is anticipated in the future. Furthermore, while VEGFR1^+^ ECs represent only a subset of the endothelial cell population, CD31 is expressed in both vascular and lymphatic ECs. Given that lymphatic vessels have been reported to contribute to the maintenance of hair follicle homeostasis^50^, future studies are expected to simultaneously observe and analyze lymphatic vessel responses in this context.

## Materials and methods

### Chemicals

MD and TST were purchased from Tokyo Chemical Industry Co., Ltd. and FUJIFILM Wako Pure Chemical Corp., respectively. Five % of MD solution was obtained by dissolving MD powder in a mixture of water, ethanol, phosphoric acid, and 1,3-butylene glycol at room temperature for 30 min using a magnetic stirrer. The vehicle consists of all the ingredients solution except for MD. The VEGF-A monoclonal antibody bevacizumab (Bev) (#HY-P9906, Avastin), anti-CCL2-neutralizing antibody (A2132)**,** and recombinant human CCL2 (#AF-300–04) were purchased from MedChemExpress Co., Ltd., Selleck Inc., and Pepro Tech, Inc., respectively.

### Mice

All experimental procedures involving mice and their care were conducted in accordance with the ARRIVE guidelines and approved by the Committee on the Ethics of Animal Experiments in Kobe Gakuin University (A23-31). Every effort was made to minimize the suffering of the mice. *VEGFR1* (*Flt1*)*-tdsRed* transgenic mice were generated by inserting a tandem dsRed reporter into the first exon of the *VEGFR1* gene within a BAC clone containing the entire *VEGFR1* locus using RED/ET recombination as previously described^51^. The modified BAC DNA was microinjected into fertilized BDF1 eggs, and two lines showing strong vascular fluorescence faithfully reproduced endogenous *VEGFR1* expression. All mice were crossed with C57BL/6 J mice more than 10 × and maintained. Wild-type mice were purchased from Japan SLC (Shizuoka, Japan).

Drug administration experiments used mice aged 8 to 12 weeks. This age corresponds to young adult mice with fully activated hair cycles. In aging studies, 2 months old mice are considered fully mature young adult. In contrast, 15 months old mice are thought to correspond to middle-aged adult humans, who typically exhibit skin aging and early alopecia commonly appear.

### Immunohistochemistry

Female C57BL/6 J mice (8–12 weeks old) were used for drug administration experiments, with an average body weight of 18.2 ± 2.5 g at the start of the experiment. For the study of age-related changes, the average body weight at the start was 34.6 ± 3.1 g for young mice (8–10 weeks old) and 45.5 ± 6.9 g for aged mice (60–63 weeks old). To synchronize the hair cycle, the dorsal hair of the mice at 2 or 15 months old during telogen was plucked. Mice were euthanized by intraperitoneal injection of a medetomidine-midazolam-butorphanol mixture (10 ml/kg). After dorsal skin samples were collected, cervical dislocation was performed to ensure death. Dorsal skin samples were treated with a microwave oven 700 w for 30 s, followed by 30 min on ice in 4% paraformaldehyde. After cryoprotection in 30% sucrose, we embedded fixed tissue in OCT compound (Sakura Tissue-Tek) and prepared sections on a cryostat. Cryostat Sects. (150 µm) were treated with blocking buffer (10% donkey serum and 0.1% Triton X-100, pH 7.4) for 1 h at room temperature, followed by incubation with primary antibodies diluted in the same buffer overnight at 4 °C. Furthermore, the sections were washed thrice with 0.1% phosphate-buffered saline with Tween® detergent (PBST) for 10 min and incubated for 1 h at room temperature with secondary antibodies. Next, we washed them again in PBST for 10 min thrice at room temperature and mounted them under a cover glass with a mounting medium. We used the following primary antibodies: GFP (rabbit, 1/1000; MBL #598), GFP (rat, 1/1000; nacalai tesque #04,404–8), VEGF (rabbit, 1/500; Abcam #ab46154), Ki67/MKI67 (rabbit, 1/500; Novus Biologicals #NB110-89,717), Keratin 15 (Chicken, 1/500; Biolegend #833,904), CD31 (rat, 1/500; BD Biosciences #557,355), CD31 (hamster, 1/200; merckmillipore #MAB1398Z), LYVE1 (rat, 1/200; Invitrogen #14–0443-82), and CCL2 (hamster, 1/100; Thermo Fisher Scientific #14–7096-81). Images were acquired on a confocal microscope (FV3000, Olympus) or a fluorescent microscope (IX81, Olympus). CellSens and Metamorph software suites were used to acquire all confocal and fluorescent microscope images, respectively. Images were processed using Adobe Photoshop.

The capillary density was quantified based on previous reports^52^. *VEGFR1*-DsRed^+^ and CD31^+^ vasculature area was defined in ImageJ as shown in Supplementary Fig. 1d, and subsequently quantified. Briefly, using DAPI staining as a morphological guide, we divided the perifollicular vessels in each image into three regions: Layer 1 [L1] (vicinity of infundibulum and isthmus) was defined as the upper area of the superior border of the bulge; Layer 2 [L2] (vicinity of Bg) was defined as characteristic slight protrusion, and further confirmed by a dense aggregation of small and intense cell nuclei; Layer 3 [L3] (vicinity of DP) was defined as the region extending from the inferior border of the bulge downward to the base of the hair follicle, encompassing the DP. Each layer was enclosed into a region of interest (ROI) by the freehand drawing tool of ImageJ. 8-bit grayscale images were generated for each channel, and a uniform threshold was applied to distinguish specific staining from background. Following threshold application, a binary mask was created where the area (pixel) of the perifollicular vessels above the threshold were calculated in total area and each layer. Because the hair cycle is accompanied by marked changes in skin area, quantification based solely on area was insufficient. Therefore, we also evaluated the perifollicular vessel ratio (%) of each layer to account for hair cycle-dependent changes, as shown in Supplementary Fig. 1b, using the following formula: Perifollicular vessel ratio (%) = Perifollicular vessel area (pixels)/Layer area (pixels) × 100. Multiple fields of view were analyzed for each replicate during measurement.

To further characterize changes in capillary vessels surrounding the DP, we analyzed the vasculature within a 100 µm diameter region centered on the DP. The DP was identified based on its characteristic morphology based on DAPI staining. Using the oval selection tool, a circular ROI with a 100 µm diameter was created and saved. The ROI was centered on the dermal papilla of each hair follicle. Within this ROI, the area occupied by VEGFR1-DsRed^+^ and CD31^+^ capillary vessels were quantified.

To define “the ratio of Ki67^+^ cells in the vicinity of vessels,” we analyzed the Z-projected images where each frame had a resolution of 512 × 512 pixels. If the distance between the Ki67^+^ cells and blood vessels was below 60 μm in the overlaid in Z-projected images, the Ki67^+^ cells were defined as “Ki67^+^ cells in the vicinity of vessels.” The Ki67^+^ cells were quantified using ImageJ on 40 × or 60 × skin fields, and only the Ki67 + cells within the hair follicle were counted. Dermal papilla size was defined by the K15-negative area and quantified using ImageJ, after the skin sections were stained with DAPI and K15.

### Cell culture ECs-derived conditioned medium (EC-CM) preparation

HUVECs were purchased from Lonza Japan and cultured at 37 °C under a 5% CO_2_ atmosphere, and were maintained in EGM-2 medium (#CC-3162, Lonza) with additive factor kit. HFDPCs were obtained from PromoCell and cultivated in Follicle Dermal Papilla Cell Basal Medium (PromoCell, Heidelberg, Germany). Both normal human dermal fibroblasts (NHDF) and human dermal lymphatic endothelial cells (HDLECs) were purchased from Takara. All experiments were conducted using cells at passages 4–6. For HUVEC subculture, trypsin/EDTA (0.025%/0.01 mM) was used. Meanwhile, HFDPCs were detached using the PromoCell Detach Kit according to the manufacturer’s instructions. To prepare ECs-derived conditioned medium (EC-CM)^51^, passage 4 (P4) HUVECs were cultured to 50%−60% confluency in a 100-mm cell culture dish in 10 mL of EGM-2 medium with or without MD for 24 h. Then, the medium was replaced with 10 mL of fresh EGM-2 medium for 24 h, and the EC-CM was harvested. After centrifugation at 3000 min^−1^ for 10 min to remove the cell debris, the EC-CM was filtered through a 0.22 μm filter (Millipore) and directly used for HFDPC culture (P4). To prepare lymphatic ECs-derived conditioned medium (LC-CM), passage 4 (P4) HDLECs were cultured to 50%−60% confluency in a 100-mm cell culture dish in 10 mL of EGM-2 medium for 24 h. The EGM-2 medium (10 mL) incubated for 24 h in a culture dish without cells was used as the control.

### Cell proliferation assay

Before the experiments, cells were seeded in 96-well plates and incubated for 24 h at 37 °C under a 5% CO_2_ atmosphere. Cells were then treated with EC-CM, ECMD-CM, or CCL2 recombinant protein for 24 h under the same culture conditions. The proliferation rate was measured using the Cell Counting Kit-8 (#343–07,623, DOJINDO) and an absorbance meter according to the manufacturer’s protocol.

### Real-time qPCR (RT-qPCR)

Total RNA was isolated using the RNeasy Mini Kit (Qiagen) in line with the manufacturer’s instructions. In addition, we synthesized cDNA from 250 ng of RNA by using a QuantiTect Retrotranscriptase reaction kit (Qiagen) and conducted qPCR by using SYBR green labeling (SYBR Premix Ex TaqII, Takara) and a TP850 Real-Time PCR System (Takara), with glyceraldehyde-3-phosphate dehydrogenase expression as the internal control. All individual sample reactions were conducted thrice. The relative fold change in target gene expression was then calculated according to the ∆∆Ct method. Supplementary Table 1 lists the qPCR primer pairs.

### RNA sequencing and data analysis

We used the Direct-Zol RNA miniprep kit (Zymo Research) and the Agilent 2200 TapeStation to extract total RNA and determine its quality, respectively. Sequencing libraries were prepared using the SMART-Seq v4 Ultra Low Input Kit for Sequencing (TaKaRa) and sequenced on an Illumina Novaseq 6000 to generate 150 bp paired-end reads. Reads were aligned using DRAGEN with the human reference genome (GENCODE/GRCh38 [hg38]). We used differentially expressed genes in GO term analysis, which then employed Expression Miner 2.0 to find enriched functional annotations.

### Tube formation assay

The procedures were performed as previously described^53^. Briefly, 48-well plates were coated with Matrigel (Corning) and incubated for 30 min at 37 °C under a 5% CO_2_ atmosphere. HUVECs were plated on the gel and cultured in the medium with or without MD, TST, and CCL2 recombinant protein. After 6 h of additional incubation, we acquired microscopic images of the tubes by using a phase-contrast microscope. Five images were then captured per well and analyzed using ImageJ software. HUVECs from passage 4 were used for this assay. All experimental conditions were replicated thrice.

### Topical treatment with MD or TST

Seven-week-old female VEGFR1-dsRed C57BL/6 J mice synchronized in the telogen stages were used. All mice were plucked at the dorsal areas using sterile forceps to synchronize hair follicle to the anagen stage. After 3 days, we then randomly grouped the animals. From this time point, one group received topical treatment with either 100 μl vehicle (1,3-Butylene Glycol/Ethanol/Purified Water) or 100 μl 5% MD (common concentration used as hair growth agent in humans) at 9 AM for 27 days to evaluate MD application, and another group received topical treatment with either 100 μl vehicle or 100 μl 0.05% TST^54^ or 100 μl 0.05% TST plus 100 μl 5% MD at 9 AM for 25 days to evaluate TST application. Sampling time points were determined based on dorsal skin color changes. In C57BL/6 mice, dorsal skin pigmentation correlates with the hair cycle, appearing dark during anagen and returning to a pink color upon entry into telogen. After the skin turned pink, treatments were continued for an additional 5 days to ensure complete entry into the telogen stage. Thereafter, mice were euthanized and the dorsal skin samples were dissected and used for immunostaining analyses.

### Administration of VEGF- and CCL2-neutralizing antibody

The 5 mg/kg Bev (concentration at 0.5 mg/ml) (#HY-P9906, MCH), VEGF-neutralizing antibody^55^, or vehicle (saline) was intraperitoneally injected during the telogen phase (P38) at 9 AM for 6 consecutive days, after which the back skin was collected for immunostaining. In this experiment, no dramatic abnormalities were observed in tissues following short-term Bev administration, and no significant difference in skin thickness was confirmed between the Bev-treated group and the control group (Supplementary Fig. 2c). To evaluate the effect of CCL2, mice were treated topically with 100 μl vehicle or 100 μl 5% MD on back skin at 9 AM for 25 consecutive days, with the intradermal administration of 100 μl vehicle (saline) or anti-CCL2-neutralizing antibody (60 μg in 100 μl, original concentration at 3.5 mg/ml) (#A2132 Selleck) every three days immediately after the application of MD (based on our pilot experiments (Supplementary Fig. 3c) and previous report^56^). Sampling time points were also determined based on dorsal skin color changes. Treatments were continued for an additional 5–7 days after the skin turned pink to ensure complete entry into the telogen stage. Thereafter, mice were euthanized, and dorsal skin samples were collected for immunostaining analyses.

### Quantification and statistical analysis

Differences between two groups were evaluated using an unpaired Student’s t-test. For comparisons among three or more groups, statistical analysis was performed using one-way analysis of variance (ANOVA). When a significant difference was detected, pairwise comparisons between groups were conducted using the Least Significant Difference (LSD) post hoc test. Data are presented as mean ± Standard Error of the Mean (SEM), and p < 0.05 was considered statistically significant.

## Supplementary Information

Supplementary Information 1.

Supplementary Information 2.

Supplementary Information 3.

Supplementary Information 4.

Supplementary Information 5.

## Data Availability

Sequence data that support the findings of this study have been deposited in the NCBI with the primary accession code GSE282648.
