# Supplementary figures and images for: Preferential crosstalk between perifollicular capillary vessels and dermal papilla cells during hair cycling homeostasis
Source: Sci Rep. 2026 Apr 1;16:15328. doi: 10.1038/s41598-026-46001-2 (PMC13181128; doi:10.1038/s41598-026-46001-2)

# Supplementary Figure.1

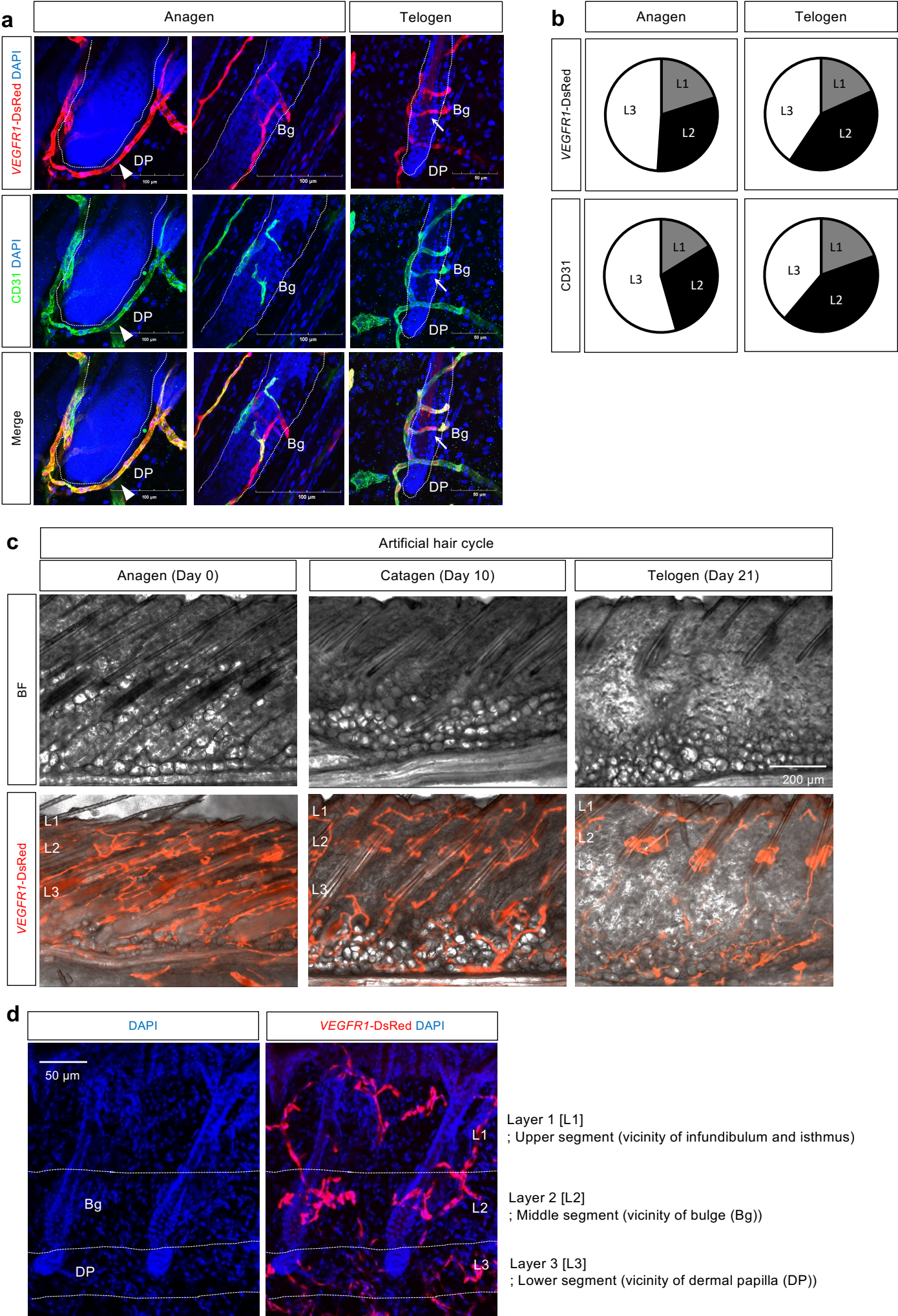

Supplement: Supplementary file 1 — Supplementary Information 1. [file 41598_2026_46001_MOESM1_ESM.pdf]

# Supplementary Figure.2

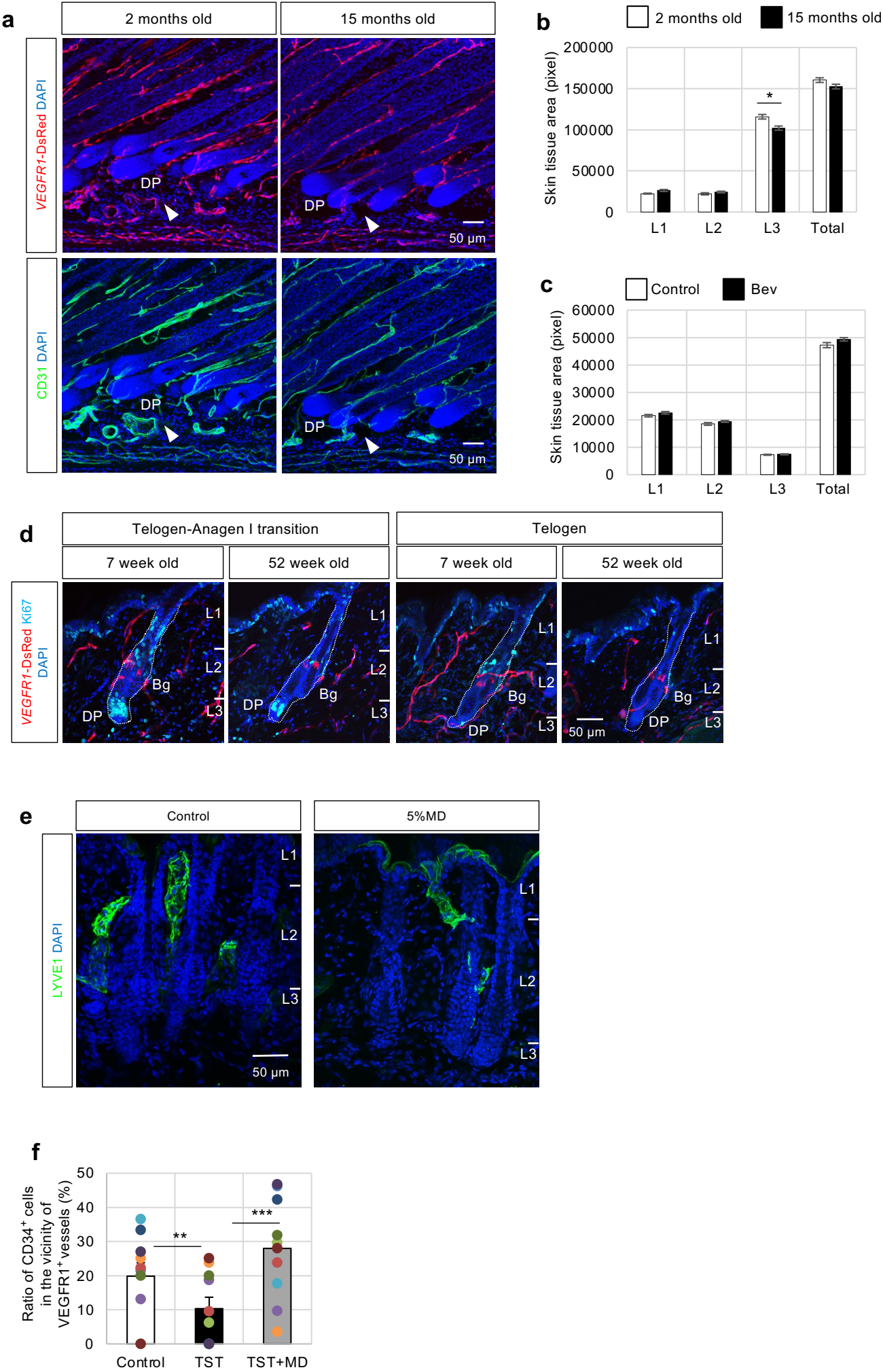

Supplement: Supplementary file 2 — Supplementary Information 2. [file 41598_2026_46001_MOESM2_ESM.pdf]

# Supplementary Figure.3

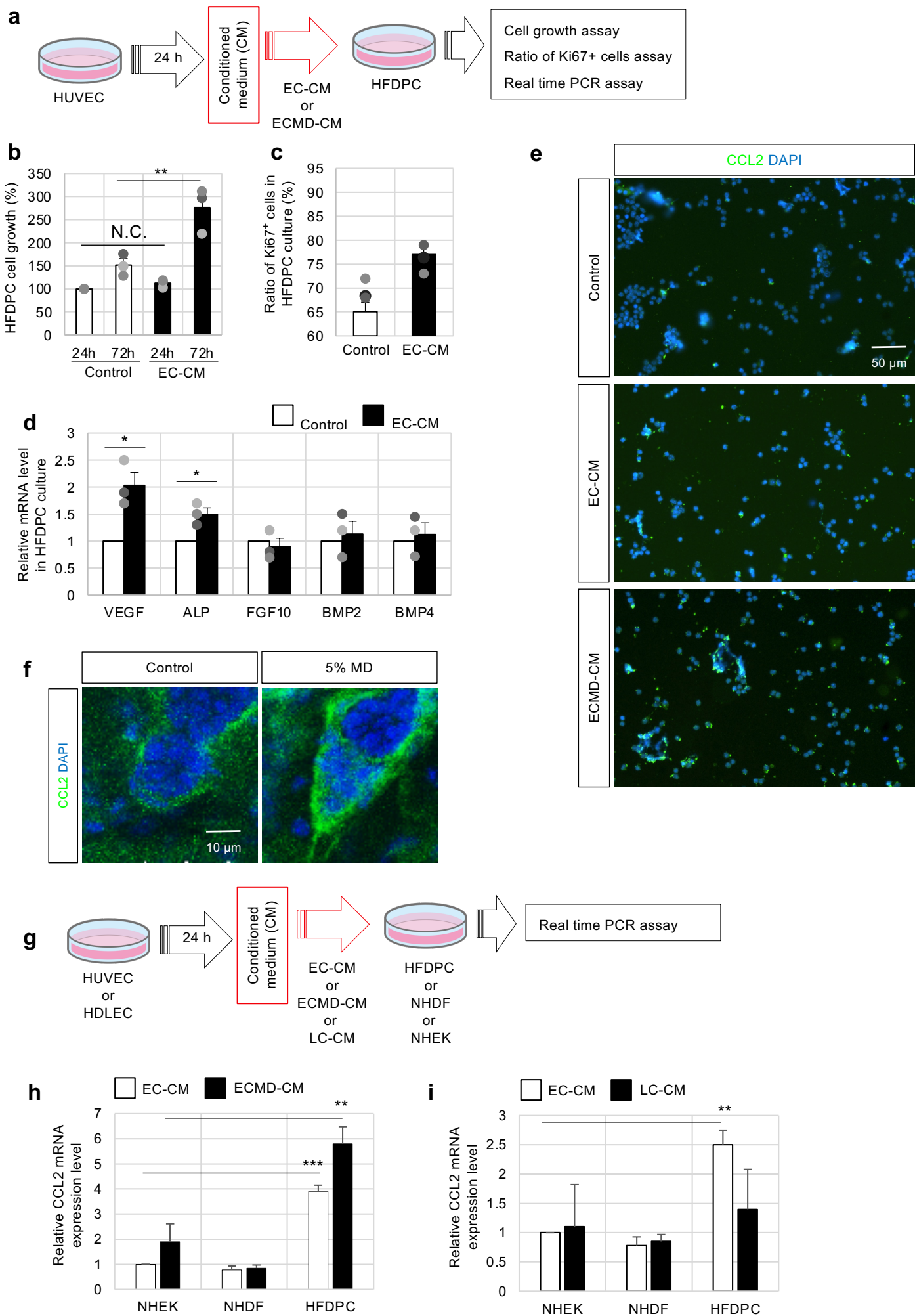

Supplement: Supplementary file 3 — Supplementary Information 3. [file 41598_2026_46001_MOESM3_ESM.pdf]
