# Supplementary material for: Preferential crosstalk between perifollicular capillary vessels and dermal papilla cells during hair cycling homeostasis
Source: Sci Rep. 2026 Apr 1;16:15328. doi: 10.1038/s41598-026-46001-2 (PMC13181128; doi:10.1038/s41598-026-46001-2)

Supplementary Figure.4

a

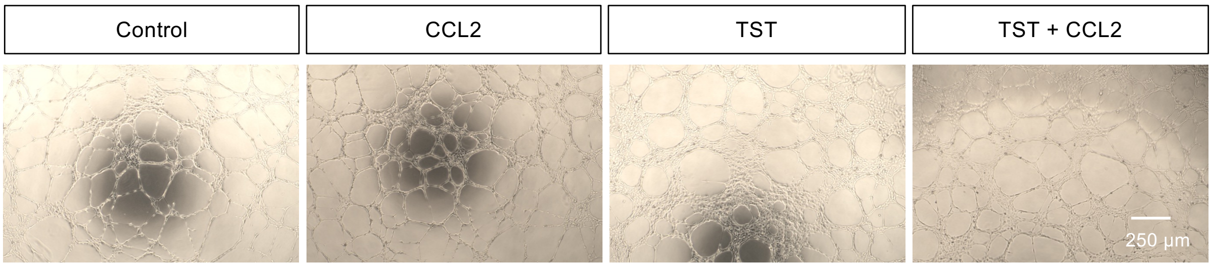

b

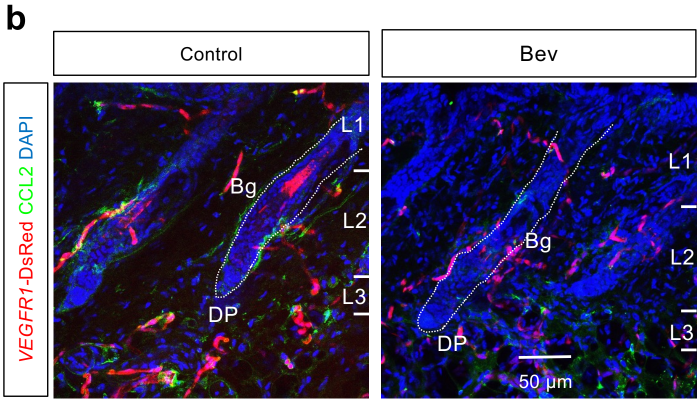

c

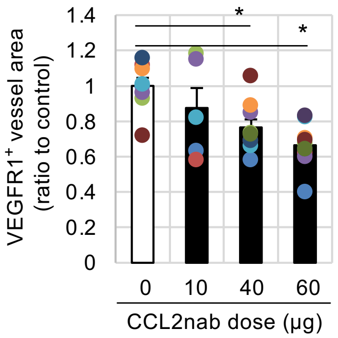

d

Quantification of CCL2 expression in Layer 3 [L3]

Group	Mean±SEM	Significant
Control	100.0±70.8	n.s.
MD	231.8±61.7	
Anagen	100.0±4.6	p<0.001
Telogen	0.5±0.1	
P2m	100.0±13.2	p<0.05
P15m	69.3±7.8	

e

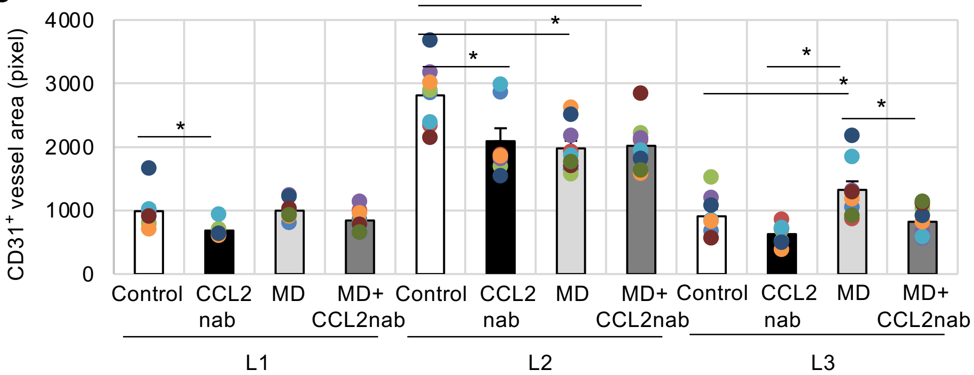

Supplement: Supplementary file 4 — Supplementary Information 4. [file 41598_2026_46001_MOESM4_ESM.pdf]
