# Supplementary material for: Preferential crosstalk between perifollicular capillary vessels and dermal papilla cells during hair cycling homeostasis
Source: Sci Rep. 2026 Apr 1;16:15328. doi: 10.1038/s41598-026-46001-2 (PMC13181128; doi:10.1038/s41598-026-46001-2)
Supplement: Supplementary file 5 — Supplementary Information 5. [file 41598_2026_46001_MOESM5_ESM.pdf]

# Supplementary Table.1

| Gene | Forward Primer | Reverse Primer |
|---|---|---|
| GAPDH | GGCCTCCAAGGAGTAAGACC | AGGGGTCTACATGGCAACTG |
| VEGF | CCTTGCTGCTCTACCTCCAC | CACACAGGATGGCTTGAAGA |
| ALP | CAAACCGAGATACAAGCACT | CGAAGAGACCCAATAGGTAG |
| FGF10 | CACATTGTGCCTCAGCCTTT | AAGAGTTGGTGGCCTCTGGT |
| BMP2 | AACGAGTGGGAAAACAACCC | GTCACGGGGAATTTCGAGTT |
| BMP4 | CACTGGCTGACCACCTCAAC | GGCACCCACATCCCTCTACT |
